# Heritability of nervous system tumors: a sibling-based design

**DOI:** 10.3389/fonc.2023.928008

**Published:** 2024-01-08

**Authors:** Giorgio Tettamanti, Ralf Kuja-Halkola, Catharina Lavebratt, Mats Talbäck, Alexander Viktorin, Michael E. Scheurer, Maria Feychting, Maral Adel Fahmideh

**Affiliations:** ^1^ Unit of Epidemiology, Institute of Environmental Medicine, Karolinska Institutet, Stockholm, Sweden; ^2^ Department of Medical Epidemiology and Biostatistics, Karolinska Institutet, Stockholm, Sweden; ^3^ Department of Molecular Medicine and Surgery, Karolinska Institutet, and Center for Molecular Medicine, Karolinska University Hospital, Stockholm, Sweden; ^4^ Department of Pediatrics, Section of Hematology-Oncology, Dan L. Duncan Comprehensive Cancer Center, Baylor College of Medicine, Houston, TX, United States; ^5^ Center for Epidemiology and Population Health, Department of Pediatrics, Baylor College of Medicine, Houston, TX, United States; ^6^ Department of Medicine, Section of Epidemiology and Population Sciences, Dan L. Duncan Comprehensive Cancer Center, Baylor College of Medicine, Houston, TX, United States

**Keywords:** heritability, nervous system tumors, sibling-based design, quantitative genetic modelling, registry-linkage study

## Abstract

**Background:**

The contribution of genetic and environmental factors to susceptibility to nervous system tumors remains unclear. We performed a quantitative genetic study using a sibling design to estimate the heritability of nervous system tumors, as well as the proportion of the risk of these tumors, which is attributable to environmental factors.

**Methods:**

We conducted a population-based cohort study using Swedish National Register data. All individuals born in Sweden during 1950–2010 with available information on both biological parents were included. A Multi-Generation Register was used to identify family clusters, including both full- and half-siblings. Initially, one index person was randomly selected from each cluster containing only full siblings and one sibling was randomly assigned to this index person. Subsequently, within each of the remaining clusters of full- and half-siblings, an index person was randomly selected, and a half-sibling was randomly assigned to this index person. Among the randomly selected siblings, cases of nervous system tumors were identified using the cancer registry. Quantitative genetic models were used to estimate the proportion of the variance in nervous system tumors attributable to additive genetic factors, shared environment, and individual-specific environment.

**Results:**

The heritability of nervous system tumors was estimated to be 29% (95% confidence interval (CI) = 19%–39%), while the contribution of the non-shared environment to the variance of nervous system tumors was estimated to be 71% (95% CI = 61%–81%). The shared environmental parameter was estimated as zero in the full model.

**Conclusion:**

The variation in susceptibility to nervous system tumors is predominantly attributable to non-shared environmental factors, followed by genetic factors.

## Introduction

Nervous system tumors are considered multifactorial traits resulting from alterations in various genes and their interactions in concert with multiple environmental factors (1). The proportion of the risk of these significant malignancies, attributable to inherited genetic and environmental factors, is poorly understood. To the best of our knowledge, few studies have evaluated the contribution of inherited genetic factors to the development of nervous system tumors. One quantitative genetic study assessed the heritability of these tumors using a family based design that included various pairs of relatives and employed structural models (2). However, the results of this study have not been replicated and are not consistent with the heritability of gliomas estimated by Genome-Wide Complex Trait Analysis (GCTA) (3). Therefore, the genetic contribution to the susceptibility of nervous system tumors remains unclear.

Familial aggregation studies are the main approach for the assessment of hereditary effects of such complex disorders and provide reliable familial risk estimates (4, 5). In addition, familial segregation studies have precisely investigated the factors responsible for familial aggregation, including the mode of inheritance and the proportion of trait risk, which is attributable to the genetic constitution within families (6, 7). However, these studies could not evaluate the overall magnitude of genetic and environmental influences on the development of complex traits, including nervous system tumors.

Family-based quantitative genetic studies are commonly used to estimate the contribution of genetic and environmental factors to susceptibility to complex traits, in which, concordance comparisons of the phenotype of interest in family settings are utilized (8). Studies of twins (monozygotic and dizygotic pairs) and siblings (full-sibling and half-sibling pairs) are the most commonly used models in genetic studies to estimate the heritability of the phenotype of interest. In a twin design, it is assumed that monozygotic and dizygotic twins share 100% and 50% of their genetic factors, respectively, and these values are assumed to be 50% for full siblings and 25% for half siblings (8).

The published pan-cancer, family based quantitative genetic studies, using a traditional twin-design, were not able to assess the heritability of nervous system tumors, due to lack of statistical power (9, 10). In the present quantitative genetic study, we estimated the contribution of genetic and environmental factors to the susceptibility to nervous system tumors in a sibling design, which is important for developing risk prediction models to prevent nervous system tumors.

## Methods

### Study subjects and procedures

#### Study population

This study is a population-based cohort study based on Swedish national register data, which contains information on individuals’ life events including birth, death, marital status, name changes, family relationships, change of sex, and place of residence. Individuals who were born in Sweden at any time during the period between 1 January 1950 and 31 December 2010 with available information on both biological parents constitute the eligible population (11, 12). This study was approved by the Ethical Review Board in Stockholm, Sweden.

#### Sibling design and case identification

The Total Population Register was linked to the Multi-Generation Register using the unique personal identification number assigned to each Swedish resident (13). The Multi-Generation Register contains the relationships between parents and their children for all individuals born in 1932 and residents of Sweden since 1960. This register has high coverage with available information on 97% of mothers and 95% of fathers of individuals and contains information such as sex, date and country of birth, number of children, date of adoption, and date of immigration (13). Siblings that share at least one parent constitute a family cluster that includes both full and half-siblings. Initially, one index person was randomly selected from each cluster containing only full siblings and one sibling was randomly assigned to this index person. Subsequently, within each of the remaining clusters of full- and half-siblings, an index person was randomly selected, and a half-sibling was randomly assigned to this index person. When creating half-siblings’ clusters, to avoid duplication among clusters, increase the number of clusters consisting of half-siblings, and boost the statistical power, we prioritized the paternal side over the maternal side, and in the general population, the number of paternal half-siblings is greater than that of maternal half siblings. Furthermore, none of the full siblings were twins, and no positive concordant twins were identified in the data.

Cases of nervous system tumors were identified through linkage of the selected siblings with the Cancer Register, using personal identification numbers (14). The Cancer Register, established in 1958, covers all cancer diagnoses in Sweden with >96% coverage (14). Cases were identified using the International Statistical Classification of Diseases and Related Health Problems, Seventh Revision (ICD-7) code 193, as this classification has been used since the start of the register, in parallel with newer classifications, to allow investigations of longer time series with consistent tumor classification. Cases diagnosed in Sweden on 1 January 1958 and 31 December 2010 were included in the study.

### Statistical analysis

We fitted a univariate model in which the variance in the nervous system tumors as the phenotype of interest was modeled to be due to additive genetic factors (A), shared environment (C), and individual-specific environment (E) (8). Full siblings and half siblings were assumed to share 50% and 25% of their additive genetic factors (A), respectively. The shared environment (C) was assumed to be fully shared among sibling pairs, regardless of whether they were full siblings or half siblings. The individual-specific environment (E) was assumed to be unique for each individual. The full model (ACE) and reduced model (AE), where the shared environment parameter (C) in the full model was estimated to be zero, were fitted to assess the heritability of the trait of interest (8).

The analyses were performed based on the liability-threshold approach and structural equation modeling (8). We assumed that the underlying liabilities were normally distributed and estimated the correlations between these underlying normal distributions using tetrachoric correlation (15). Prevalence was adjusted for birth year, assuming that it has a linear effect. In a sensitivity analysis, we compared the estimated concordance rates and tetrachoric correlations based on the dataset that was used for the quantitative genetic analyses to those obtained from a different dataset that included all full- and half-sibling pairs prior to random selection of individuals. The technical terms utilized are defined as follows: affected concordant = sibling pairs diagnosed with nervous system tumors; non-affected concordant = sibling pairs without any nervous system tumor diagnosis; discordant = sibling pairs with only one sibling diagnosed with nervous system tumors. SAS statistical software version 9.3 (SAS Institute, Inc., Cary, NC, USA) was used to prepare the data, and the mets-package (16) in R version 3.3.3 (cran.r-project.org) was used to conduct the analyses.

## Results

In total, 6,266,266 persons with information on both biological parents constituted the eligible population, and 7,023,749 possible sibling pairs were identified. **Table 1** summarizes the demographic characteristics of all possible sibling pairs in the eligible population. From this population, we identified 3,338,980 family clusters, including 2,790,830 full-sibling pairs, 182,157 maternal half-sibling pairs, and 365,993 paternal half-sibling pairs. From the identified family clusters, 1,748,528 full sibling pairs, 117,298 maternal half-sibling pairs, and 226,196 paternal half-sibling pairs were randomly selected, of which 25, 1, and 1 were positive concordant pairs. The demographic characteristics of the randomly selected siblings and positive concordant pairs are summarized in **Table 2**.

**Table 1:** Descriptive characteristics of full siblings and half-siblings in the population.

	Full siblings			Maternal half-siblings			Paternal half-siblings		
	Affected Concordant	Non-affected Concordant	Discordant	Affected Concordant	Non-affected Concordant	Discordant	Affected Concordant	Non-affected Concordant	Discordant
**Number of siblings**	53	4127789	15185	4	1260820	3668	8	1611074	5148
Sex, n (%)									
Same-sex male	12 (22.6)	1098694 (26.6)	4125 (27.2)	0 (0.0)	330542 (26.2)	952 (26.0)	0 (0.0)	420434 (26.1)	1288 (25.0)
Same-sex female	14 (26.4)	978243 mn(23.7)	3503 (23.1)	0 (0.0)	299838 (23.8)	870 (23.7)	2 (25.0)	386364 (24.0)	1294 (25.1)
Different sex	27 (50.9)	2050852 (49.7)	7557 (49.8)	4 (100.0)	630440 (50.0)	1846 (50.3)	6 (75.0)	804276 (49.9)	2566 (49.8)
Birth year, n (%)									
1950-1959	15 (28.3)	403601 (9.8)	3226 (21.2)	0 (0.0)	30104 (2.4)	232 (6.3)	0 (0.0)	41122 (2.5)	326 (6.3)
1960-1969	7 (13.2)	422974 (10.2)	2222 (14.6)	0 (0.0)	38832 (3.1)	208 (5.7)	0 (0.0)	56574 (3.5)	320 (6.2)
1970-1979	3 (5.7)	351580 (8.5)	1365 (9.0)	2 (50.0)	44604 (3.5)	180 (4.9)	0 (0.0)	50940 (3.2)	176 (3.4)
1980-1989	4 (7.5)	389729 (9.4)	1032 (6.8)	0 (0.0)	51158 (4.1)	124 (3.4)	0 (0.0)	51046 (3.2)	102 (1.9)
1990-1999	2 (3.8)	418183 (10.1)	684 (4.5)	0 (0.0)	58188 (4.6)	82 (2.2)	0 (0.0)	54900 (3.4)	106 (2.1)
2000-2010	0 (0.0)	461022 (11.2)	281 (1.9)	0 (0.0)	57970 (4.6)	24 (0.7)	0 (0.0)	56546 (3.5)	26 (0.5)
Different	22 (41.5)	1680703 (40.7)	6375 (42.0)	2 (50.0)	979964 (77.7)	2818 (76.8)	8 (100.0)	1299946 (80.7)	4092 (79.5)

**Table 2:** Descriptive characteristics of full siblings and half-siblings included in the study.

	Full siblings			Maternal half-siblings			Paternal half-siblings		
	Affected Concordant	Non-affected Concordant	Discordant	Affected Concordant	Non-affected Concordant	Discordant	Affected Concordant	Non-affected Concordant	Discordant
**Number of siblings**	25	1742569	5934	1	116967	330	1	225525	670
Sex, n (%)									
Same-sex male	3 (12.0)	453506 (26.0)	1562 (26.3)	0 (0.0)	30548 (26.1)	91 (27.6)	0 (0.0)	59132 (26.2)	170 (25.4)
Same-sex female	8 (32.0)	404258 (23.2)	1357 (22.9)	0 (0.0)	27606 (23.6)	78 (23.6)	1 (100.0)	53930 (23.9)	175 (26.1)
Different sex	14 (56.0)	884805 (50.8)	3015 (50.8)	1 (100.0)	58813 (50.3)	161 (48.8)	0 (00)	112463 (49.9)	325 (48.5)
Birth year, n (%)									
1950-1959	9 (36.0)	179601 (10.3)	1407 (23.7)	0 (0.0)	3163 (2.7)	24 (7.3)	0 (0.0)	6007 (2.7)	43 (6.4)
1960-1969	4 (16.0)	180377 (10.4)	897 (15.1)	0 (0.0)	2915 (2.5)	25 (7.6)	0 (0.0)	7672 (3.4)	46 (6.9)
1970-1979	1 (4.0)	169690 (9.7)	594 (10.0)	0 (0.0)	3694 (3.2)	12 (3.6)	0 (0.0)	7298 (3.2)	25 (3.7)
1980-1989	2 (8.0)	153292 (8.8)	382 (6.4)	0 (0.0)	4079 (3.5)	15 (4.6)	0 (0.0)	6520 (2.9)	13 (1.9)
1990-1999	2 (8.0)	191475 (11.0)	253 (4.3)	0 (0.0)	5398 (4.6)	8 (2.4)	0 (0.0)	7717 (3.4)	18 (2.7)
2000-2010 Different	0 (0.0) 7 (28.0)	265729 (15.2) 602405 (34.6)	144 (2.4) 2257 (38.1)	0 (0.0) 1 (100.0)	8841 (7.6) 88877 (75.9)	4 (1.2) 242 (73.3)	0 (0.0) 1 (100.0)	12079 (5.4) 178232 (79.0)	8 (1.2) 517 (77.2)

As **Table 3** illustrates, in the full model (ACE), the heritability of nervous system tumors was estimated at 28% (95% confidence interval (CI) = 19%–38%), while the contribution of non-shared environment in the variance of nervous system tumors was estimated to be 71% (95% CI = 62%–81%). The shared environment parameter (C) in the full model was estimated to be zero. Therefore, an AE model was fitted in which the C-parameter was set to zero. In the AE model, the heritability of nervous system tumors was estimated to be 29% (95% CI = 19%–39%), with the remaining variance attributable to the non-shared environment at 71% (95% CI = 61%–81%).

**Table 3:** Heritability estimate of nervous system tumors using a sibling design.

		Estimated Variance (95% CI)		
Model		Additive Genetic (A)	Shared Environment (C)	Non-shared Environment (E)
ACE		0.28 (0.19-0.38)	0.00 (-0.00-0.01)	0.71 (0.62-0.81)
**Tetrachoric correlations**				
	**Rho**	**SE**	**Positive concurrent pairs**	
Full siblings	0.18	0.03	25	
Maternal half-siblings	0.15	0.12	1	
Paternal half-siblings	0.07	0.11	1	
AE		0.29 (0.19-0.39)	NA	0.71(0.61-0.81)
**Tetrachoric correlations**				
	**Rho**	**SE**	**Positive concurrent pairs**	
Full siblings	0.18	0.03	25	
Maternal half-siblings	0.15	0.12	1	
Paternal half-siblings	0.07	0.11	1	

In a sensitivity analysis, we observed that the concordance rates in the dataset used for assessing heritability (0.008 for full siblings and 0.004 for half siblings) were similar to those estimated when using all sibling pairs prior to random selection of individuals (0.007 for full siblings and 0.003 for half siblings). Additionally, among randomly selected pairs, an almost two times higher tetrachoric correlation was observed for full siblings compared to half siblings (0.18 compared to 0.10), which was similar to the difference found between all full and half-sibling pairs prior to random selection (0.14 for full siblings, 0.06, half siblings).

## Discussion

This quantitative genetic study with a sibling design estimated the heritability of nervous system tumors at 29%. Additionally, in this study, non-shared environmental factors accounted for 71% of the variation in susceptibility to nervous system tumors. These findings are in line with the general notion that nervous system tumors, similar to most neoplasms, are predominantly attributable to environmental factors and somatic aberrations (17, 18).

To our knowledge, this is the largest and most comprehensive family based quantitative genetic study of nervous system tumors. The heritability of nervous system tumors, along with several other cancer types, has been previously evaluated in a family based quantitative genetic study and estimated at 12% (2). The study was designed based on various pairs of relatives, and cancer cases were identified from the Swedish Cancer Register during 1958–1996 (2). Our study included cases diagnosed in Sweden during 1958–2010 and employed recently developed algorithms for assessing heritability in quantitative genetic studies. Furthermore, the environmental and heritable risk factors of various cancer types, including brain and other nervous system tumors, were evaluated based on the Nordic Twin registers, using a traditional twin design (9, 10). However, due to a small number of identified positive concordant pairs and subsequently insufficient statistical power, also after longer follow-up (9), they did not proceed with assessing the heritability of nervous system tumors.

Additionally, one study used GCTA to assess the proportion of variance in glioma attributable to genetic factors (3). GCTA estimates the heritability of a trait by simultaneous evaluation of all single nucleotide polymorphisms (SNPs), contributing to a genome-wide association study (GWAS). GCTA transforms the estimated heritability to the liability scale using disease prevalence. The estimated array-based heritability of glioma is 25%, of which ~30% is explained by currently identified variants and 70% remains to be identified (3, 19). Hence, our finding that inherited genes contribute 29% to the total risk of developing nervous system tumors is in line with the estimated array-based heritability of glioma.

We estimated the contribution of inherited genetic factors to the causation of nervous system tumors at 29%, of which a small fraction is attributable to the single-gene mutations of rare genetic syndromes; a small proportion is explained by variants currently identified by genetic association studies, and most of the genetic constitution remains unexplained, indicating a major gap in our understanding of heritable nervous system tumors (19).

The prevalence of germline mutations in cancer-predisposing genes among children and adolescents with cancer is estimated to be approximately 8% (20). Recently, a pan-cancer study reported that of the investigated nervous system tumors (including high-grade gliomas, atypical teratoid rhabdoid tumors, SHH medulloblastomas, and retinoblastomas), 20% of cases are associated with a known pathogenic germline variant (162 genes investigated) (21). Since the investigated germline variants constitute a small fraction of genetic factors predisposing individuals to nervous system tumors, most genetic variations underlying nervous system tumorigenesis have yet to be identified.

Further, the results were generated based on Swedish national register data and were thus mainly applicable to Caucasian ancestry. We assumed that heritability differs slightly across various ancestries, a hypothesis that can be tested in other populations. Additionally, heritability differs slightly among different histotypes, although it is difficult to predict trends. Given the identified results, clinical genetic consultation might be beneficial for first-degree relatives of patients diagnosed with nervous system tumors, considering that even among families with hereditary syndromes predisposing to nervous system tumors, the histological subtypes of the tumors might vary.

This study has several strengths. To date, our study represents the largest family based quantitative genetic study performed on nervous system tumors. Population-based register data were used to identify family relationships, family clusters, and cases of nervous system tumors that limited selection and recall bias. Additionally, identifying cases within family clusters through the nationwide Cancer Register provided a unique opportunity to investigate these rare tumors in many family clusters with sufficient statistical power. This study had some limitations. It is known that hereditary effects are strongest in early onset cancers (2, 22). The heritability estimated in this study was based on pooled cases of pediatric and adult nervous system tumors. Thus, one of the limitations of this study is that we could not report nervous system tumor heritability stratified by age because of the rarity of these tumors and the lack of sufficient statistical power. However, we adjusted our analyses for birth year and aimed to measure the overall heritability for all ages of nervous system tumor onset. In addition, owing to limited statistical power, we could not measure the heritability of different histotypes of nervous system tumors. Thus, extended studies including more family clusters, and consequently more sibling pairs and more positive concordant pairs, would be needed in the future to measure the heritability of nervous system tumors in pediatric and adult cases as well as different histotypes independently. In addition, while performing this study based on a sibling design, rather than a traditional twin design, provided enough positive concordant pairs overall, the number of positive concordant pairs was small among half-siblings. However, in a sensitivity analysis, we found that the tetrachoric correlations in full siblings were two times greater than the tetrachoric correlations in half-siblings in both randomly selected pairs (the pairs included in the main analyses) as well as in all sibling pairs before random selection, which indicates a higher similarity among full siblings compared to half-siblings, supporting the hypothesis of genetic contribution to the occurrence of nervous system tumors.

In conclusion, the variation in susceptibility to nervous system tumors was predominantly attributable to non-shared environmental factors (71%), followed by inherited genetic factors (29%). Although the effect of inherited genes on the total risk of developing nervous system tumors is more pronounced among children, adults and children may share some common inherited genetic factors (23). The genetic architecture of nervous system tumors and the environmental exposures involved in the development of these tumors are to be explored. Incorporating genetic and environmental factors in concert with somatic characteristics of tumors is essential for a better understanding of nervous system tumorigenesis and the development of risk prediction models (19).

## Data availability statement

The original contributions presented in the study are included in the article/supplementary material. Further inquiries can be directed to the corresponding author.

## Ethics statement

The study was approved by the Ethical Review Board in Stockholm, Sweden. As the study is based on the registry data, consent from the participants was waived. The study was performed in accordance with the Declaration of Helsinki.

## Author contributions

MF and MAF have designed the study. GT, RK-H, MT, and MAF have performed the analyses. GT, RK-H, AV, MS, and MAF have interpreted the results. All authors have been involved in writing the manuscript. All authors contributed to the article and approved the submitted version.
